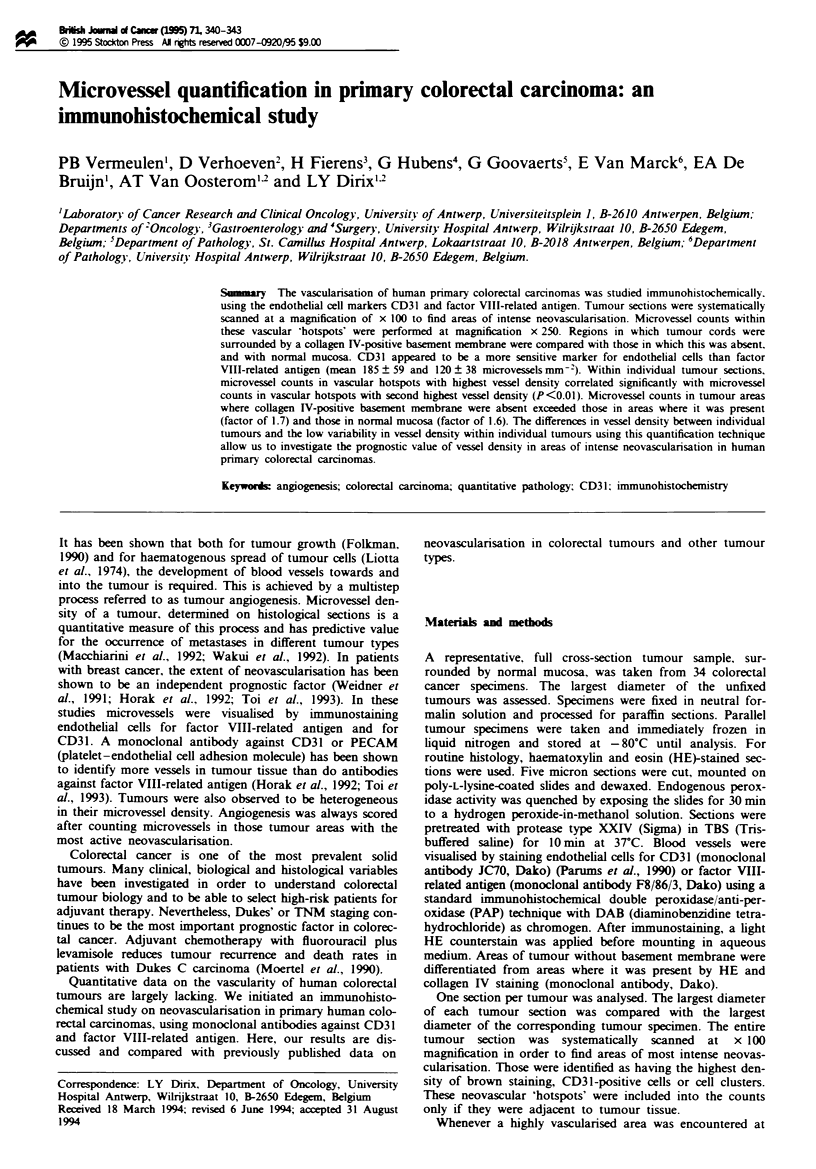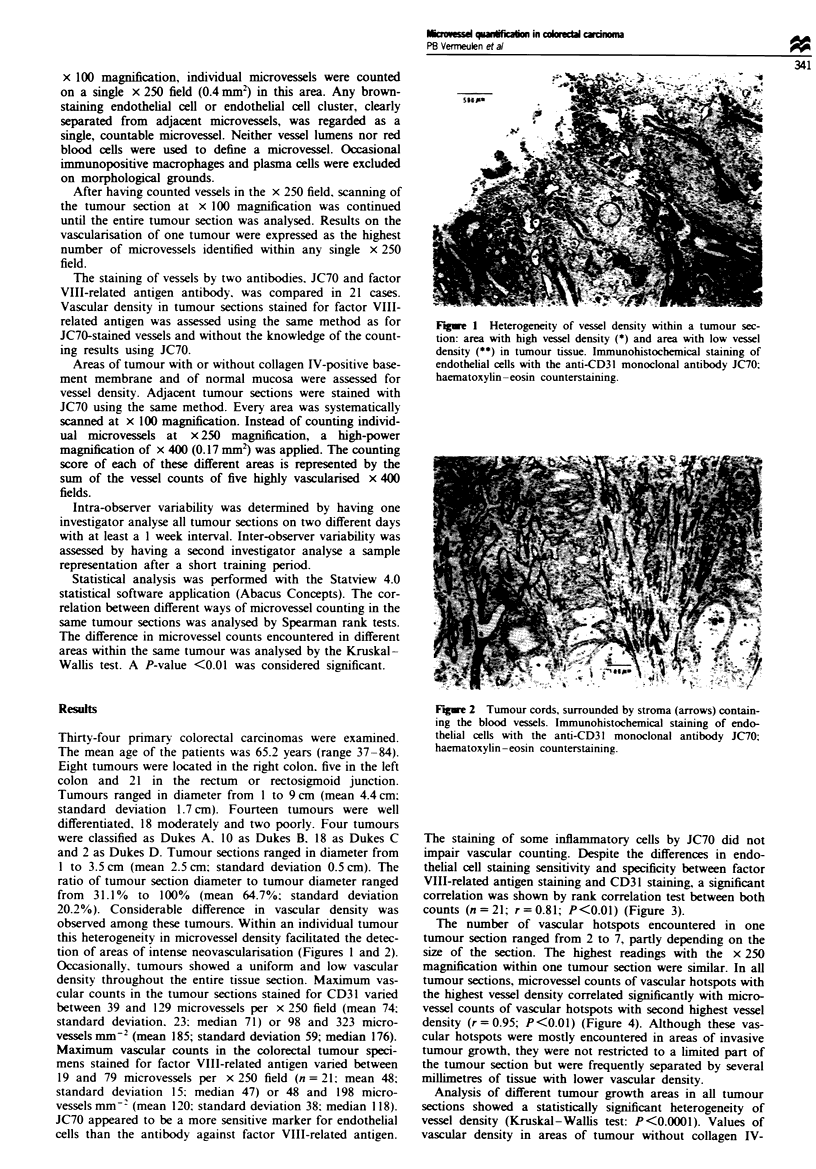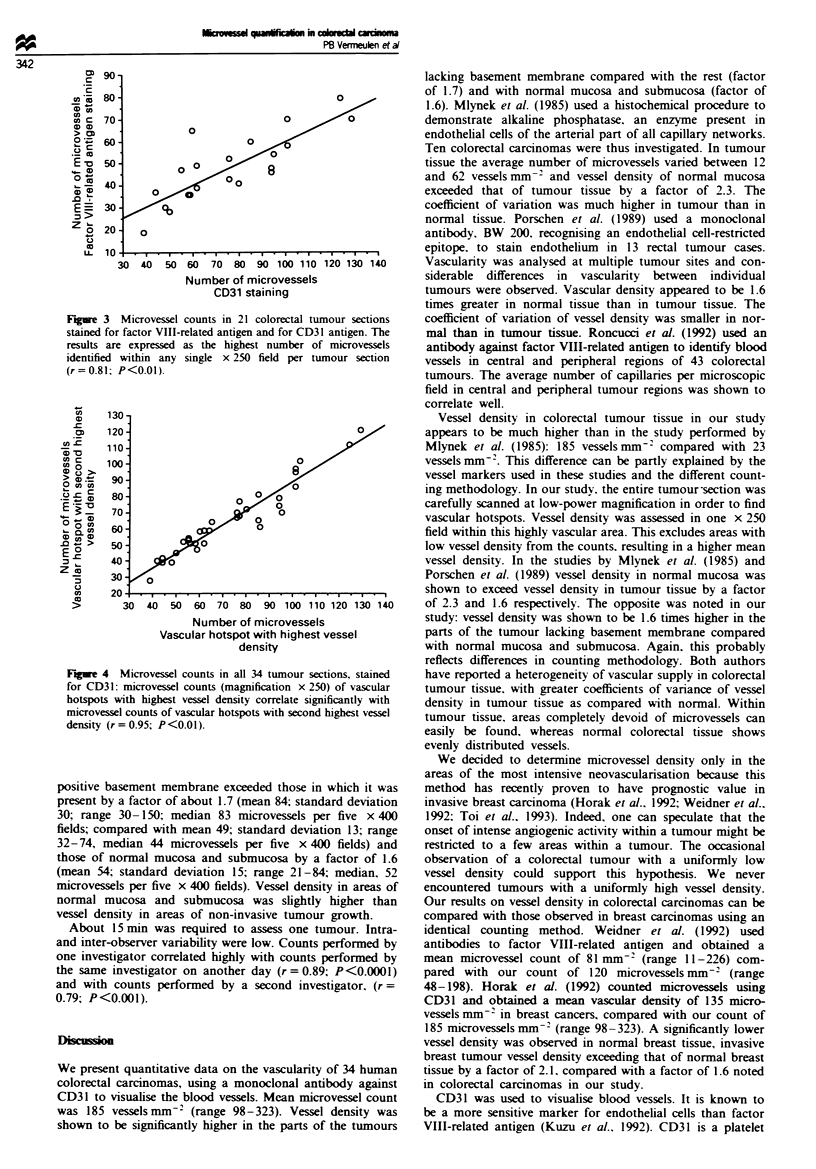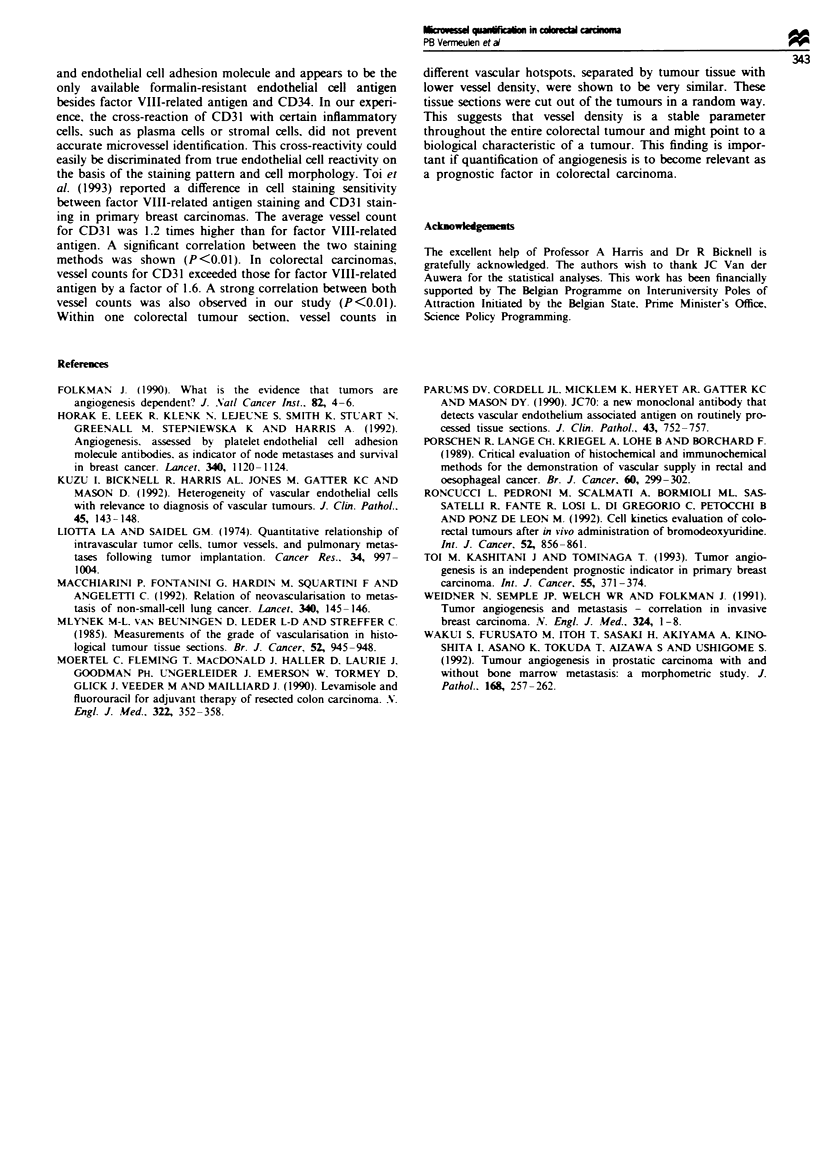# Microvessel quantification in primary colorectal carcinoma: an immunohistochemical study.

**DOI:** 10.1038/bjc.1995.68

**Published:** 1995-02

**Authors:** P. B. Vermeulen, D. Verhoeven, H. Fierens, G. Hubens, G. Goovaerts, E. Van Marck, E. A. De Bruijn, A. T. Van Oosterom, L. Y. Dirix

**Affiliations:** Laboratory of Cancer Research and Clinical Oncology, University of Antwerp, Belgium.

## Abstract

**Images:**


© 1995 Stockton Press All rights reserved 0007-0920/95 $9.00

### Microvessel quantification in primary colorectal carcinoma: an immunohistochemical study

PB Vermeulen[1], D Verhoeven[2], H Fierens[3], G Hubens[4], G Goovaerts[5], E Van Marck[6], EA De Bruijn[1], AT Van Oosterom[1,2] and LY Dirix[1,2]

[1]*Laboratory of Cancer Research and Clinical Oncology, University of Antwerp, Universiteitsplein 1, B-2610 Antwerpen, Belgium; Departments of* [2]*Oncology,* [3]*Gastroenterology and* [4]*Surgery, University Hospital Antwerp, Wilrijkstraat 10, B-2650 Edegem, Belgium;* [5]*Department of Pathology, St. Camillus Hospital Antwerp, Lokaartstraat 10, B-2018 Antwerpen, Belgium;* [6]*Department of Pathology, University Hospital Antwerp, Wilrijkstraat 10, B-2650 Edegem, Belgium.*

**Summary** The vascularisation of human primary colorectal carcinomas was studied immunohistochemically, using the endothelial cell markers CD31 and factor VIII-related antigen. Tumour sections were systematically scanned at a magnification of × 100 to find areas of intense neovascularisation. Microvessel counts within these vascular 'hotspots' were performed at magnification × 250. Regions in which tumour cords were surrounded by a collagen IV-positive basement membrane were compared with those in which this was absent, and with normal mucosa. CD31 appeared to be a more sensitive marker for endothelial cells than factor VIII-related antigen (mean 185 ± 59 and 120 ± 38 microvessels $mm^{-2}$). Within individual tumour sections, microvessel counts in vascular hotspots with highest vessel density correlated significantly with microvessel counts in vascular hotspots with second highest vessel density ($P<0.01$). Microvessel counts in tumour areas where collagen IV-positive basement membrane were absent exceeded those in areas where it was present (factor of 1.7) and those in normal mucosa (factor of 1.6). The differences in vessel density between individual tumours and the low variability in vessel density within individual tumours using this quantification technique allow us to investigate the prognostic value of vessel density in areas of intense neovascularisation in human primary colorectal carcinomas.

**Keywords:** angiogenesis; colorectal carcinoma; quantitative pathology; CD31; immunohistochemistry

It has been shown that both for tumour growth (Folkman, 1990) and for haematogenous spread of tumour cells (Liotta *et al.*, 1974), the development of blood vessels towards and into the tumour is required. This is achieved by a multistep process referred to as tumour angiogenesis. Microvessel density of a tumour, determined on histological sections is a quantitative measure of this process and has predictive value for the occurrence of metastases in different tumour types (Macchiarini *et al.*, 1992; Wakui *et al.*, 1992). In patients with breast cancer, the extent of neovascularisation has been shown to be an independent prognostic factor (Weidner *et al.*, 1991; Horak *et al.*, 1992; Toi *et al.*, 1993). In these studies microvessels were visualised by immunostaining endothelial cells for factor VIII-related antigen and for CD31. A monoclonal antibody against CD31 or PECAM (platelet-endothelial cell adhesion molecule) has been shown to identify more vessels in tumour tissue than do antibodies against factor VIII-related antigen (Horak *et al.*, 1992; Toi *et al.*, 1993). Tumours were also observed to be heterogeneous in their microvessel density. Angiogenesis was always scored after counting microvessels in those tumour areas with the most active neovascularisation.

Colorectal cancer is one of the most prevalent solid tumours. Many clinical, biological and histological variables have been investigated in order to understand colorectal tumour biology and to be able to select high-risk patients for adjuvant therapy. Nevertheless, Dukes' or TNM staging continues to be the most important prognostic factor in colorectal cancer. Adjuvant chemotherapy with fluorouracil plus levamisole reduces tumour recurrence and death rates in patients with Dukes C carcinoma (Moertel *et al.*, 1990).

Quantitative data on the vascularity of human colorectal tumours are largely lacking. We initiated an immunohistochemical study on neovascularisation in primary human colorectal carcinomas, using monoclonal antibodies against CD31 and factor VIII-related antigen. Here, our results are discussed and compared with previously published data on neovascularisation in colorectal tumours and other tumour types.

Correspondence: LY Dirix, Department of Oncology, University Hospital Antwerp, Wilrijkstraat 10, B-2650 Edegem, Belgium
Received 18 March 1994; revised 6 June 1994; accepted 31 August 1994

### Materials and methods

A representative, full cross-section tumour sample, surrounded by normal mucosa, was taken from 34 colorectal cancer specimens. The largest diameter of the unfixed tumours was assessed. Specimens were fixed in neutral formalin solution and processed for paraffin sections. Parallel tumour specimens were taken and immediately frozen in liquid nitrogen and stored at −80°C until analysis. For routine histology, haematoxylin and eosin (HE)-stained sections were used. Five micron sections were cut, mounted on poly-L-lysine-coated slides and dewaxed. Endogenous peroxidase activity was quenched by exposing the slides for 30 min to a hydrogen peroxide-in-methanol solution. Sections were pretreated with protease type XXIV (Sigma) in TBS (Tris-buffered saline) for 10 min at 37°C. Blood vessels were visualised by staining endothelial cells for CD31 (monoclonal antibody JC70, Dako) (Parums *et al.*, 1990) or factor VIII-related antigen (monoclonal antibody F8/86/3, Dako) using a standard immunohistochemical double peroxidase anti-peroxidase (PAP) technique with DAB (diaminobenzidine tetrahydrochloride) as chromogen. After immunostaining, a light HE counterstain was applied before mounting in aqueous medium. Areas of tumour without basement membrane were differentiated from areas where it was present by HE and collagen IV staining (monoclonal antibody, Dako).

One section per tumour was analysed. The largest diameter of each tumour section was compared with the largest diameter of the corresponding tumour specimen. The entire tumour section was systematically scanned at × 100 magnification in order to find areas of most intense neovascularisation. Those were identified as having the highest density of brown staining, CD31-positive cells or cell clusters. These neovascular 'hotspots' were included into the counts only if they were adjacent to tumour tissue.

Whenever a highly vascularised area was encountered at

× 100 magnification, individual microvessels were counted on a single × 250 field (0.4 $mm^2$) in this area. Any brown-staining endothelial cell or endothelial cell cluster, clearly separated from adjacent microvessels, was regarded as a single, countable microvessel. Neither vessel lumens nor red blood cells were used to define a microvessel. Occasional immunopositive macrophages and plasma cells were excluded on morphological grounds.

After having counted vessels in the × 250 field, scanning of the tumour section at × 100 magnification was continued until the entire tumour section was analysed. Results on the vascularisation of one tumour were expressed as the highest number of microvessels identified within any single × 250 field.

The staining of vessels by two antibodies, JC70 and factor VIII-related antigen antibody, was compared in 21 cases. Vascular density in tumour sections stained for factor VIII-related antigen was assessed using the same method as for JC70-stained vessels and without the knowledge of the counting results using JC70.

Areas of tumour with or without collagen IV-positive basement membrane and of normal mucosa were assessed for vessel density. Adjacent tumour sections were stained with JC70 using the same method. Every area was systematically scanned at × 100 magnification. Instead of counting individual microvessels at × 250 magnification, a high-power magnification of × 400 (0.17 $mm^2$) was applied. The counting score of each of these different areas is represented by the sum of the vessel counts of five highly vascularised × 400 fields.

Intra-observer variability was determined by having one investigator analyse all tumour sections on two different days with at least a 1 week interval. Inter-observer variability was assessed by having a second investigator analyse a sample representation after a short training period.

Statistical analysis was performed with the Statview 4.0 statistical software application (Abacus Concepts). The correlation between different ways of microvessel counting in the same tumour sections was analysed by Spearman rank tests. The difference in microvessel counts encountered in different areas within the same tumour was analysed by the Kruskal–Wallis test. A $P$-value $<0.01$ was considered significant.

### Results

Thirty-four primary colorectal carcinomas were examined. The mean age of the patients was 65.2 years (range 37–84). Eight tumours were located in the right colon, five in the left colon and 21 in the rectum or rectosigmoid junction. Tumours ranged in diameter from 1 to 9 cm (mean 4.4 cm; standard deviation 1.7 cm). Fourteen tumours were well differentiated, 18 moderately and two poorly. Four tumours were classified as Dukes A, 10 as Dukes B, 18 as Dukes C and 2 as Dukes D. Tumour sections ranged in diameter from 1 to 3.5 cm (mean 2.5 cm; standard deviation 0.5 cm). The ratio of tumour section diameter to tumour diameter ranged from 31.1% to 100% (mean 64.7%; standard deviation 20.2%). Considerable difference in vascular density was observed among these tumours. Within an individual tumour this heterogeneity in microvessel density facilitated the detection of areas of intense neovascularisation (Figures 1 and 2). Occasionally, tumours showed a uniform and low vascular density throughout the entire tissue section. Maximum vascular counts in the tumour sections stained for CD31 varied between 39 and 129 microvessels per × 250 field (mean 74; standard deviation, 23; median 71) or 98 and 323 microvessels $mm^{-2}$ (mean 185; standard deviation 59; median 176). Maximum vascular counts in the colorectal tumour specimens stained for factor VIII-related antigen varied between 19 and 79 microvessels per × 250 field ($n = 21$; mean 48; standard deviation 15; median 47) or 48 and 198 microvessels $mm^{-2}$ (mean 120; standard deviation 38; median 118). JC70 appeared to be a more sensitive marker for endothelial cells than the antibody against factor VIII-related antigen.

**Figure 1** Heterogeneity of vessel density within a tumour section: area with high vessel density (*) and area with low vessel density (**) in tumour tissue. Immunohistochemical staining of endothelial cells with the anti-CD31 monoclonal antibody JC70; haematoxylin–eosin counterstaining.

**Figure 2** Tumour cords, surrounded by stroma (arrows) containing the blood vessels. Immunohistochemical staining of endothelial cells with the anti-CD31 monoclonal antibody JC70; haematoxylin–eosin counterstaining.

The staining of some inflammatory cells by JC70 did not impair vascular counting. Despite the differences in endothelial cell staining sensitivity and specificity between factor VIII-related antigen staining and CD31 staining, a significant correlation was shown by rank correlation test between both counts ($n = 21$; $r = 0.81$; $P<0.01$) (Figure 3).

The number of vascular hotspots encountered in one tumour section ranged from 2 to 7, partly depending on the size of the section. The highest readings with the × 250 magnification within one tumour section were similar. In all tumour sections, microvessel counts of vascular hotspots with the highest vessel density correlated significantly with microvessel counts of vascular hotspots with second highest vessel density ($r = 0.95$; $P<0.01$) (Figure 4). Although these vascular hotspots were mostly encountered in areas of invasive tumour growth, they were not restricted to a limited part of the tumour section but were frequently separated by several millimetres of tissue with lower vascular density.

Analysis of different tumour growth areas in all tumour sections showed a statistically significant heterogeneity of vessel density (Kruskal–Wallis test: $P<0.0001$). Values of vascular density in areas of tumour without collagen IV-

**Figure 3** Microvessel counts in 21 colorectal tumour sections stained for factor VIII-related antigen and for CD31 antigen. The results are expressed as the highest number of microvessels identified within any single $\times 250$ field per tumour section ($r = 0.81$; $P<0.01$).

**Figure 4** Microvessel counts in all 34 tumour sections, stained for CD31: microvessel counts (magnification $\times 250$) of vascular hotspots with highest vessel density correlate significantly with microvessel counts of vascular hotspots with second highest vessel density ($r = 0.95$; $P<0.01$).

positive basement membrane exceeded those in which it was present by a factor of about 1.7 (mean 84; standard deviation 30; range 30–150; median 83 microvessels per five $\times 400$ fields; compared with mean 49; standard deviation 13; range 32–74, median 44 microvessels per five $\times 400$ fields) and those of normal mucosa and submucosa by a factor of 1.6 (mean 54; standard deviation 15; range 21–84; median, 52 microvessels per five $\times 400$ fields). Vessel density in areas of normal mucosa and submucosa was slightly higher than vessel density in areas of non-invasive tumour growth.

About 15 min was required to assess one tumour. Intra- and inter-observer variability were low. Counts performed by one investigator correlated highly with counts performed by the same investigator on another day ($r = 0.89$; $P<0.0001$) and with counts performed by a second investigator, ($r = 0.79$; $P<0.001$).

### Discussion

We present quantitative data on the vascularity of 34 human colorectal carcinomas, using a monoclonal antibody against CD31 to visualise the blood vessels. Mean microvessel count was 185 vessels $mm^{-2}$ (range 98–323). Vessel density was shown to be significantly higher in the parts of the tumours lacking basement membrane compared with the rest (factor of 1.7) and with normal mucosa and submucosa (factor of 1.6). Mlynek *et al.* (1985) used a histochemical procedure to demonstrate alkaline phosphatase, an enzyme present in endothelial cells of the arterial part of all capillary networks. Ten colorectal carcinomas were thus investigated. In tumour tissue the average number of microvessels varied between 12 and 62 vessels $mm^{-2}$ and vessel density of normal mucosa exceeded that of tumour tissue by a factor of 2.3. The coefficient of variation was much higher in tumour than in normal tissue. Porschen *et al.* (1989) used a monoclonal antibody, BW 200, recognising an endothelial cell-restricted epitope, to stain endothelium in 13 rectal tumour cases. Vascularity was analysed at multiple tumour sites and considerable differences in vascularity between individual tumours were observed. Vascular density appeared to be 1.6 times greater in normal tissue than in tumour tissue. The coefficient of variation of vessel density was smaller in normal than in tumour tissue. Roncucci *et al.* (1992) used an antibody against factor VIII-related antigen to identify blood vessels in central and peripheral regions of 43 colorectal tumours. The average number of capillaries per microscopic field in central and peripheral tumour regions was shown to correlate well.

Vessel density in colorectal tumour tissue in our study appears to be much higher than in the study performed by Mlynek *et al.* (1985): 185 vessels $mm^{-2}$ compared with 23 vessels $mm^{-2}$. This difference can be partly explained by the vessel markers used in these studies and the different counting methodology. In our study, the entire tumour section was carefully scanned at low-power magnification in order to find vascular hotspots. Vessel density was assessed in one $\times 250$ field within this highly vascular area. This excludes areas with low vessel density from the counts, resulting in a higher mean vessel density. In the studies by Mlynek *et al.* (1985) and Porschen *et al.* (1989) vessel density in normal mucosa was shown to exceed vessel density in tumour tissue by a factor of 2.3 and 1.6 respectively. The opposite was noted in our study: vessel density was shown to be 1.6 times higher in the parts of the tumour lacking basement membrane compared with normal mucosa and submucosa. Again, this probably reflects differences in counting methodology. Both authors have reported a heterogeneity of vascular supply in colorectal tumour tissue, with greater coefficients of variance of vessel density in tumour tissue as compared with normal. Within tumour tissue, areas completely devoid of microvessels can easily be found, whereas normal colorectal tissue shows evenly distributed vessels.

We decided to determine microvessel density only in the areas of the most intensive neovascularisation because this method has recently proven to have prognostic value in invasive breast carcinoma (Horak *et al.*, 1992; Weidner *et al.*, 1992; Toi *et al.*, 1993). Indeed, one can speculate that the onset of intense angiogenic activity within a tumour might be restricted to a few areas within a tumour. The occasional observation of a colorectal tumour with a uniformly low vessel density could support this hypothesis. We never encountered tumours with a uniformly high vessel density. Our results on vessel density in colorectal carcinomas can be compared with those observed in breast carcinomas using an identical counting method. Weidner *et al.* (1992) used antibodies to factor VIII-related antigen and obtained a mean microvessel count of 81 $mm^{-2}$ (range 11–226) compared with our count of 120 microvessels $mm^{-2}$ (range 48–198). Horak *et al.* (1992) counted microvessels using CD31 and obtained a mean vascular density of 135 microvessels $mm^{-2}$ in breast cancers, compared with our count of 185 microvessels $mm^{-2}$ (range 98–323). A significantly lower vessel density was observed in normal breast tissue, invasive breast tumour vessel density exceeding that of normal breast tissue by a factor of 2.1, compared with a factor of 1.6 noted in colorectal carcinomas in our study.

CD31 was used to visualise blood vessels. It is known to be a more sensitive marker for endothelial cells than factor VIII-related antigen (Kuzu *et al.*, 1992). CD31 is a platelet and endothelial cell adhesion molecule and appears to be the only available formalin-resistant endothelial cell antigen besides factor VIII-related antigen and CD34. In our experience, the cross-reaction of CD31 with certain inflammatory cells, such as plasma cells or stromal cells, did not prevent accurate microvessel identification. This cross-reactivity could easily be discriminated from true endothelial cell reactivity on the basis of the staining pattern and cell morphology. Toi *et al.* (1993) reported a difference in cell staining sensitivity between factor VIII-related antigen staining and CD31 staining in primary breast carcinomas. The average vessel count for CD31 was 1.2 times higher than for factor VIII-related antigen. A significant correlation between the two staining methods was shown ($P<0.01$). In colorectal carcinomas, vessel counts for CD31 exceeded those for factor VIII-related antigen by a factor of 1.6. A strong correlation between both vessel counts was also observed in our study ($P<0.01$). Within one colorectal tumour section, vessel counts in different vascular hotspots, separated by tumour tissue with lower vessel density, were shown to be very similar. These tissue sections were cut out of the tumours in a random way. This suggests that vessel density is a stable parameter throughout the entire colorectal tumour and might point to a biological characteristic of a tumour. This finding is important if quantification of angiogenesis is to become relevant as a prognostic factor in colorectal carcinoma.

**Acknowledgements**

The excellent help of Professor A Harris and Dr R Bicknell is gratefully acknowledged. The authors wish to thank JC Van der Auwera for the statistical analyses. This work has been financially supported by The Belgian Programme on Interuniversity Poles of Attraction Initiated by the Belgian State, Prime Minister's Office, Science Policy Programming.

### References

FOLKMAN J. (1990). What is the evidence that tumors are angiogenesis dependent? *J. Natl Cancer Inst.*, **82**, 4-6.

HORAK E, LEEK R, KLENK N, LEJEUNE S, SMITH K, STUART N, GREENALL M, STEPNIEWSKA K AND HARRIS A. (1992). Angiogenesis, assessed by platelet endothelial cell adhesion molecule antibodies, as indicator of node metastases and survival in breast cancer. *Lancet*, **340**, 1120-1124.

KUZU I, BICKNELL R, HARRIS AL, JONES M, GATTER KC AND MASON D. (1992). Heterogeneity of vascular endothelial cells with relevance to diagnosis of vascular tumours. *J. Clin. Pathol.*, **45**, 143-148.

LIOTTA LA AND SAIDEL GM. (1974). Quantitative relationship of intravascular tumor cells, tumor vessels, and pulmonary metastases following tumor implantation. *Cancer Res.*, **34**, 997-1004.

MACCHIARINI P, FONTANINI G, HARDIN M, SQUARTINI F AND ANGELETTI C. (1992). Relation of neovascularisation to metastasis of non-small-cell lung cancer. *Lancet*, **340**, 145-146.

MLYNEK M-L, VAN BEUNINGEN D, LEDER L-D AND STREFFER C. (1985). Measurements of the grade of vascularisation in histological tumour tissue sections. *Br. J. Cancer*, **52**, 945-948.

MOERTEL C, FLEMING T, MACDONALD J, HALLER D, LAURIE J, GOODMAN PH, UNGERLEIDER J, EMERSON W, TORMEY D, GLICK J, VEEDER M AND MAILLIARD J. (1990). Levamisole and fluorouracil for adjuvant therapy of resected colon carcinoma. *N. Engl. J. Med.*, **322**, 352-358.

PARUMS DV, CORDELL JL, MICKLEM K, HERYET AR, GATTER KC AND MASON DY. (1990). JC70: a new monoclonal antibody that detects vascular endothelium associated antigen on routinely processed tissue sections. *J. Clin. Pathol.*, **43**, 752-757.

PORSCHEN R, LANGE CH, KRIEGEL A, LOHE B AND BORCHARD F. (1989). Critical evaluation of histochemical and immunochemical methods for the demonstration of vascular supply in rectal and oesophageal cancer. *Br. J. Cancer*, **60**, 299-302.

RONCUCCI L, PEDRONI M, SCALMATI A, BORMIOLI ML, SASSATELLI R, FANTE R, LOSI L, DI GREGORIO C, PETOCCHI B AND PONZ DE LEON M. (1992). Cell kinetics evaluation of colorectal tumours after *in vivo* administration of bromodeoxyuridine. *Int. J. Cancer*, **52**, 856-861.

TOI M, KASHITANI J AND TOMINAGA T. (1993). Tumor angiogenesis is an independent prognostic indicator in primary breast carcinoma. *Int. J. Cancer*, **55**, 371-374.

WEIDNER N, SEMPLE JP, WELCH WR AND FOLKMAN J. (1991). Tumor angiogenesis and metastasis – correlation in invasive breast carcinoma. *N. Engl. J. Med.*, **324**, 1-8.

WAKUI S, FURUSATO M, ITOH T, SASAKI H, AKIYAMA A, KINOSHITA I, ASANO K, TOKUDA T, AIZAWA S AND USHIGOME S. (1992). Tumour angiogenesis in prostatic carcinoma with and without bone marrow metastasis: a morphometric study. *J. Pathol.*, **168**, 257-262.